# A tutorial of diverse genome analysis tools found in the CoGe web-platform using *Plasmodium* spp. as a model

**DOI:** 10.1093/database/bay030

**Published:** 2018-04-03

**Authors:** Andreina I Castillo, Andrew D L Nelson, Asher K Haug-Baltzell, Eric Lyons

**Affiliations:** 1 BIO5 Institute, University of Arizona, 1657 E Helen St, Tucson, AZ 85719, USA; 2 School of Plant Sciences, University of Arizona, 1140 E South Campus Dr, Tucson, AZ 85721, USA; 3 Arizona Biological/Biomedical Sciences Program, University of Arizona, Tucson, AZ 85721, USA

## Abstract

Integrated platforms for storage, management, analysis and sharing of large quantities of omics data have become fundamental to comparative genomics. CoGe (https://genomevolution.org/coge/) is an online platform designed to manage and study genomic data, enabling both data- and hypothesis-driven comparative genomics. CoGe’s tools and resources can be used to organize and analyse both publicly available and private genomic data from any species. Here, we demonstrate the capabilities of CoGe through three example workflows using 17 *Plasmodium* genomes as a model. *Plasmodium* genomes present unique challenges for comparative genomics due to their rapidly evolving and highly variable genomic AT/GC content. These example workflows are intended to serve as templates to help guide researchers who would like to use CoGe to examine diverse aspects of genome evolution. In the first workflow, trends in genome composition and amino acid usage are explored. In the second, changes in genome structure and the distribution of synonymous (Ks) and non-synonymous (Kn) substitution values are evaluated across species with different levels of evolutionary relatedness. In the third workflow, microsyntenic analyses of multigene families’ genomic organization are conducted using two *Plasmodium*-specific gene families—serine repeat antigen, and cytoadherence-linked asexual gene—as models. In general, these example workflows show how to achieve quick, reproducible and shareable results using the CoGe platform. We were able to replicate previously published results, as well as leverage CoGe’s tools and resources to gain additional insight into various aspects of *Plasmodium* genome evolution. Our results highlight the usefulness of the CoGe platform, particularly in understanding complex features of genome evolution.

**Database URL**: https://genomevolution.org/coge/

## Introduction

During the last decade, ‘omics’ data generation and collection has grown exponentially and contains valuable information about most groups in the tree of life (1–3). ‘Omics’ data are generated in laboratories around the world, and generally requires multiple tools and databases to analyse and host increasingly larger amounts of information. The difficulty of navigating this plethora of publicly available data can hinder collaborative efforts. Hence, platforms capable of leveraging large quantities of omics data, tools for its exploration and analysis and resources to facilitate reproducible and collaborative research are essential in comparative genomics. CoGe (https://genomevolution.org/coge/) is one of several platforms developed to fill this niche. The CoGe platform combines a variety of interconnected data management, analysis and visualization tools to facilitate exploratory and hypothesis-driven research of complex omics data. Though applicable to any biological group, here we showcase the types of comparative analyses that can be performed with CoGe’s tools and services by using *Plasmodium* genomes as a model.

Advances in high-throughput technologies and a desire to better understand parasites of the genus *Plasmodium*, the causal agents of malaria in humans, lead to a dramatic increase in publicly available information for the genus (4). *Plasmodium* genomes are characterized by a combination of gene loss and the acquisition of species- or lineage-specific genes, many of which mediate host–parasite interactions (5). All *Plasmodium* species have a complex life cycle involving a vertebrate host and a mosquito vector. The genomes of *Plasmodium* parasites are small (between 17 and 28 Mb) in comparison to those of their vertebrate (1 Gb for birds; 2–3 Gb for mammals) and mosquito (230–284 Mb) hosts. *Plasmodium* parasites also have shared genomic characteristics (e.g. chromosome number, an apicoplast and a mitochondria) (6). Moreover, in comparison to other groups (e.g. plant genomes), their structural organization and gene content are largely conserved across species. Nonetheless, despite these conserved features, *Plasmodium* species exhibit significant genomic sequence evolution and different *Plasmodium* clades have highly dissimilar DNA GC content. Overall, these characteristics make *Plasmodium* parasites a unique group for comparative genomic studies.

Arguably the two most important repositories for malaria research are NCBI/Genbank (7) and PlasmoDB (8). However, these platforms are somewhat limited in the ways that they allow users to interact with their data. Here, we have imported all available *Plasmodium* genomes and annotations into CoGe and made them publicly available. By making these genomes publicly available within the platform, genomic analyses beyond the scope of this tutorial can be developed *in situ* by interested researchers. All evolutionary and genomic analyses presented here were performed using CoGe’s tools and services, with links to regenerate them. Three model workflows are presented to showcase the usefulness of CoGe in different aspects of comparative genomics: (i) an assessment of compositional bias and amino acid usage, (ii) an evaluation of the frequency and location of chromosomal rearrangements through whole genome syntenic analyses, and synonymous and non-synonymous substitution trends between genomes and (iii) an exploration into the microsyntenic genomic structural differences in genus-specific multigene families.

## The CoGe web-based platform

### System requirements

CoGe is an open-access analysis platform that only requires a web browser (Chrome or Firefox are recommended) and a connection to the Internet. For full operability Flash, Javascript, popups and cookies need to be allowed.

### Genome data used on these tutorials

Representative genomes from the four major *Plasmodium* clades (simian, rodents, *Laverania* subgenus and birds/reptiles) were obtained from NCBI/Genbank (7), PlasmoDB (8) and GeneDB (9). Reference genome sequences and annotations were imported and made publicly available within the CoGe platform for usage and analysis. All publicly available *Plasmodium* genomes used in this study were organized into a CoGe Notebook: (https://genomevolution.org/coge/NotebookView.pl?lid=2155). Notebooks provide the means to manage collections of genomic, functional genomic (e.g. transcriptomic) and diversity (e.g. SNP) data. Additionally, a summary table with a list of species, CoGe’s genome IDs, their respective genome links in CoGe, their associated publication or bioproject and their in-text reference has been provided for all species referenced in the three workflows later (Supplementary File S1). In addition to using already loaded datasets, researchers may also add their own genomes or related data into CoGe. User-loaded data can be kept private, shared with collaborators or made fully public.

### Describing CoGe’s capabilities with example workflows

CoGe has a variety of analysis and visualization tools that can assist in unraveling the evolutionary histories of complex genomes. In the three workflows later, we have outlined step-by-step instructions for addressing key aspects of genome evolution, as well as a brief discussion of the insights gained from each analysis. Links to regenerate all analyses are provided in Supplementary File S2. Although all of the analyses and data may be used anonymously, researchers who log into CoGe get additional features such as automatic tracking of analyses (with links to regenerate them), the ability to add new data (can be made public and private) and access to restricted data (when permission is granted).

### Workflow 1: assessment of genome compositional bias and amino acid usage

**Figure 1.**
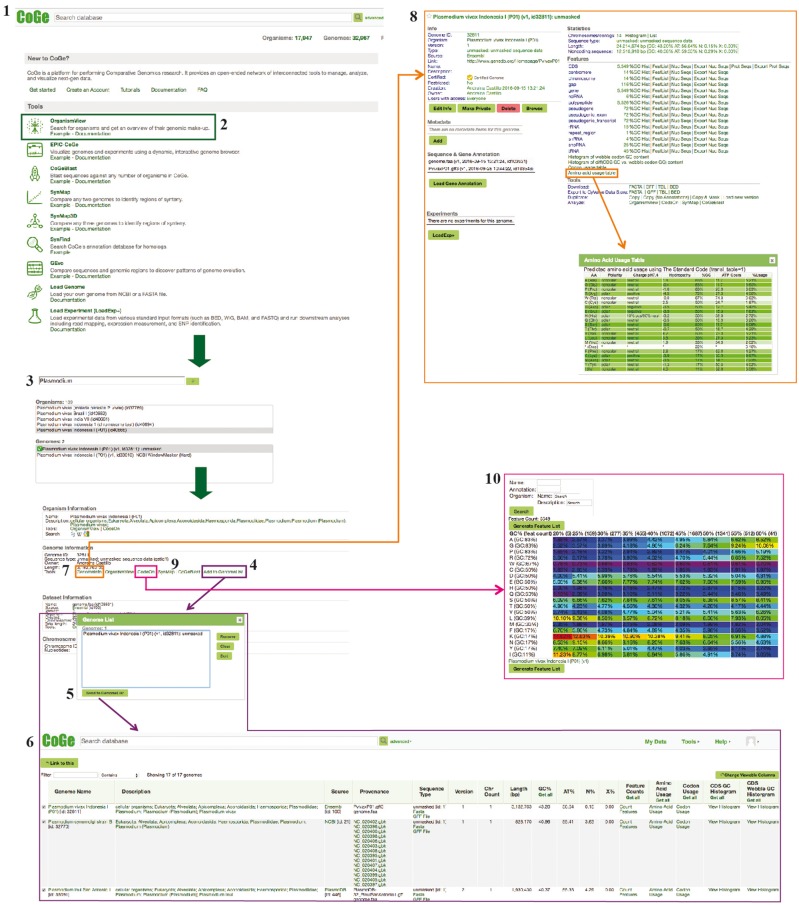
Example Workflow 1. The displayed numbers match the steps indicated in the workflow section of the text. Colors represent the different tools used in the example Workflow 1: GenomeInfo (orange), CodeOn (pink) and GenomeList (purple). Links to regenerate these screen captures are provided within the step-by-step instructions found in the text.

**Figure 2.**
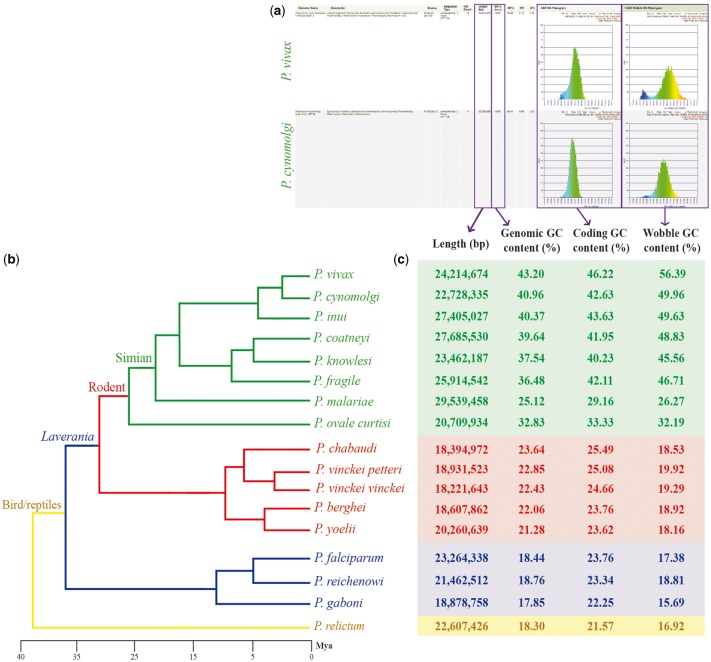
Genomic features across sequenced *Plasmodium* species from four different clades. (**a**) screen capture of GenomeList analysis (https://genomevolution.org/r/ts41) showing statistics on two genomes including histograms of CDS GC content and third nucleotide position GC content; (**b**) cladogram of *Plasmodium* species with colors demarking different clades: simian clade (green), rodent clade (red), subgenus *Laverania* (blue) and bird/reptile clade (yellow) and (**c**) table of genomic features for each *Plasmodium* species. Links to regenerate these analyses are in Supplementary File S2.

Genome nucleotide composition (i.e. GC content) has been shown to significantly affect codon and amino acid usage patterns in eukaryotes (10–12). Furthermore, GC content variations also affect chromosome length (13), gene conversion rates (14) and protein expression (15). One of the most noticeable characteristics of *Plasmodium* parasites is their variable range of genomic compositions [e.g. *Plasmodium falciparum* (18.44%) (16) and *Plasmodium vivax* (44.87%) (17)]. Thus, the degree in which changes in genome composition affect amino acid usage can be explored in detail by using *Plasmodium* parasites as models. Three CoGe’s tools—GenomeList, GenomeInfo and CodeOn—were used to characterize genome composition and its effect on amino acid usage for 17 *Plasmodium* species. A diagram of the steps followed in this example workflow is included in Figure 1. Genomic attributes for each species are shown in Figure 2, organized by their phylogeny (18).

#### How to use GenomeList, GenomeInfo and CodeOn

1. In your web-browser, navigate to CoGe (https://genomevolution.org).
2. Under ‘Tools’, click on OrganismView (https://genomevolution.org/coge/OrganismView.pl).
3. Type the scientific name of interest in the ‘Search’ box and select a genome version. Type ‘*P. vivax* Indonesia I’ and search, select the genome version ‘*P. vivax* Indonesia I (P01) (v1, id32811): unmasked’ (https://genomevolution.org/coge/OrganismView.pl?gid=32811).
4. Select ‘Add to GenomeList’ under the ‘Genome Information’ section. The selected genome will appear in a pop out window named ‘Genome List’. To add additional genomes, select a new organism and/or genome version, and click on ‘Add to GenomeList’ without closing the ‘Genome List’ window.
5. In the Genome List popup, click ‘Send to GenomeList’ to generate a table of the genomic features and attributes for each selected genome (https://genomevolution.org/r/ts41). The number of display columns can be modified with the column selection tool.
6. Different genomic features can be calculated by clicking on the corresponding column. For average genomic GC content click on ‘Get GC’. For coding sequence (CDS), GC and third nucleotide position in the codon GC content click on ‘Get all’ in the respective columns. These genomes can be downloaded by choosing the ‘Send selected genomes to’ option at the bottom of the screen, and clicking ‘Go’.
7. To examine additional genomic features of each individual genome, return to OrganismView and click on ‘GenomeInfo’ under the ‘Genome Information’ category.
8. To obtain a full list of a genome’s genomic features, select the ‘Click on Features’ option under the ‘Features’ menu. Each feature has additional options that can be further explored. For example, amino acid usage can be examined for any given genome by selecting ‘Amino acid usage table’ from the list of options (https://genomevolution.org/coge/GenomeInfo.pl?gid=32811). Predicted amino acid usage under the standard genetic code will be displayed as a table in a pop out window. This table will also contain a summary of amino acid polarity, charge, hydropathy, %GC and ATP cost to assist with interpretation. To download genome sequences (FASTA) or annotations files (GFF), select the corresponding option from ‘Downloads’ in the ‘Tools’ menu.
9. To examine the distribution of coding %GC per amino acid, return to OrganismView and click on ‘CodeOn’.
10. An amino acid usage table, binned by the overall %GC of each CDS, will be generated. The relative percent usage of any amino acid for that %GC bin will be color-coded according to the percentage usage of all other cells on the table (red, for cells with the highest usage respect to other cells; and purple, for cells with the lowest usage respect to other cells) (https://genomevolution.org/coge/CodeOn.pl?dsgid=32811). Note that due to its intensive computational nature users need to login into CoGe to use CodeOn.

#### Workflow 1 results

In a single analysis, we quickly replicated previous reports (19) showing that species closely related to *P. falciparum* (subgenus *Laverania*) have AT-rich genomes (Figure 2, blue box). Additionally, we observed increased genomic GC content in *Plasmodium* species of the rodent clade (21.28–23.64%; Figure 2, red box) (20, 21), and even higher GC values in species closely related to *P. vivax* (25.12–40.96%; Figure 2, green box) (22–25). Genomic GC content values have been independently reported for *Plasmodium* species, with only two studies (26, 27) presenting genomic GC content across several species. Nonetheless, to our knowledge, no other study has thoroughly compared GC content variation or done so in as many species as the ones included here.

We simultaneously assessed inter- and intra-clade variations of GC content in both the entire codon and specifically on the third nucleotide position. In our *Plasmodium* model, GC content in the entire codon and the third nucleotide position were strongly GC biased in GC-rich genomes and strongly AT biased in AT-rich genomes (Figure 2). Nonetheless, we identified species where GC content in the third nucleotide position was less GC biased than coding GC content (e.g. *Plasmodium malariae* and *Plasmodium ovale curtisi*). These differences were only evident by performing simultaneous multispecies comparisons. Though small, they may suggest unique long-term evolutionary trends of *P. malariae* and *P. ovale curtisi* with respect to other primate-infecting *Plasmodium* species from the simian clade.

**Figure 3.**
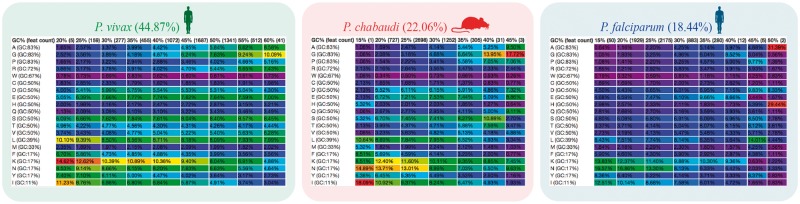
Amino acid usage table binned by the GC content of each CDS. CodeOn results for three *Plasmodium* species are shown: *P. vivax* (green; https://genomevolution.org/coge/CodeOn.pl?dsgid=32811), *Plasmodium chabaudi* (red; https://genomevolution.org/coge/CodeOn.pl?dsgid=32902) and *P. falciparum* (blue; https://genomevolution.org/coge/CodeOn.pl?dsgid=19306). Color-code indicates percent usage compared with all cells in the table (purple < blue < green < yellow < orange < red) Links to obtain amino acid usage values for each species are in Supplementary File S2.

CodeOn clearly showed a change in amino acid usage trends across species with different coding GC content (Figure 3). Amino acids at the ends of the GC composition spectrum (those coded by codons that are GC-rich or GC-poor) had the biggest change in usage across species, while amino acids in the middle of the spectrum (∼50% GC-rich) showed little to no preference (Figure 3). Despite similarities in amino acid usage, differences in the way these amino acids are coded (codon usage bias) have been reported, even in comparisons between closely related species (*Plasmodium vivax* vs. *Plasmodium knowlesi*) (28).

### Workflow 2: whole genome comparisons, synonymous (ks) and non-synonymous (kn) substitutions

Genome organizational changes have significant implications in coordinated gene expression (29), genome-specific specialization, (30) and the discovery of orthologous genes (31). CoGe’s provides powerful tools for exploring changes in genome structure and sequence evolution across multiple species, and making inferences on the evolutionary mechanisms and forces behind them.

#### SynMap

**Figure 4.**
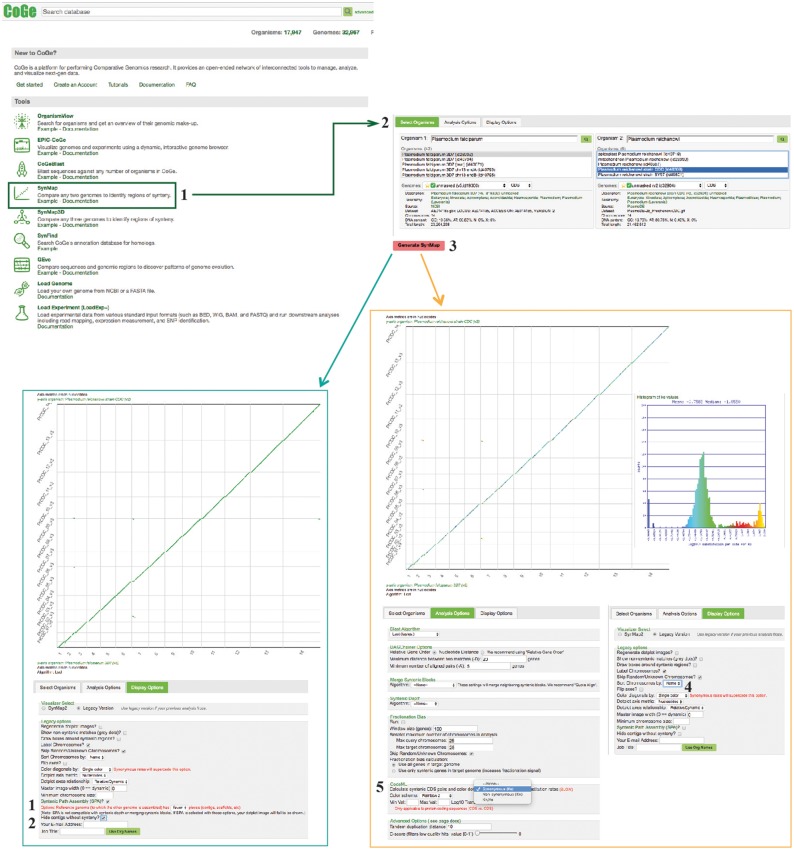
SynMap, CodeML and SPA sections of example Workflow 2. The displayed numbers match the steps indicated in the workflow section of the text. Colors represent two of the SynMap tools used on the example Workflow 2: SynMap’s SPA tool (teal) and SynMap’s CodeML tool (orange). Links to regenerate these screen captures are provided within the step-by-step instructions found in the text.

SynMap (32, 33) was used to identify large-scale changes in genome organization amongst *Plasmodium* species (Supplementary File S2). Specifically, whole genome pairwise comparisons were performed using default SynMap parameters across species pairs with different levels of evolutionary relatedness (i.e. sister taxa, closely related species and distantly related species). Briefly, SynMap (i) identifies putative syntenic gene pairs using a sequence comparison algorithm (LAST by default), (ii) identifies and filters tandem duplicated using a program called blast2raw and (iii) uses DAGChainer to find collinear series of homologous genes or sequences and identify syntenic pairs. SynMap uses CodeML (34) to calculate the non-synonymous (Kn) and synonymous (Ks) substitution rates for all syntenic gene pairs identified in each pairwise comparison, which can then be used to draw further evolutionary conclusions such as age of duplication events and acting selection. Briefly, CodeML’s workflow in CoGe is to (i) identify syntenic gene pair, (ii) extract out DNA CDS, (iii) translate to protein sequence, (iv) perform a global sequence alignment of the protein sequence using the Needleman–Wunsch global sequence alignment algorithm (https://pypi.python.org/pypi/nwalign/) and the BLOSOM62 scoring matrix, (v) back-translate the protein alignment to a codon alignment and (vi) feed the codon alignment into CodeML for Kn, Ks estimation. This workflow is detailed in the documentation for SynMap (https://goo.gl/L2XVZE). A diagram of the steps followed in this example workflow is included in Figure 4.

#### How to use SynMap

1. Find SynMap on CoGe’s main page (https://genomevolution.org/CoGe/SynMap.pl).
2. Search for and select the desired genome for Organisms 1 and 2 [e.g. type *Plasmodium* in the ‘Search’ box for Organism 1 and select *P. falciparum* 3D7 (v5, id19306), select *Plasmodium reichenowi* strain CDC (v2, id32904) for Organism 2].
3. Click on ‘Generate SynMap’ to run the analysis.
4. There are additional SynMap features to change the visualization of the dot plot. To change the main visualizer, select the Display Option tab and click on ‘Visualizer Select’. Use SynMap2, to dynamically zoom-in or -out of specific dotplot regions (https://genomevolution.org/r/wi4i). Use SynMap Legacy, to access additional visualizing options. For example, to order chromosomes by name in the dot plot, find the Legacy options menu and select ‘Sort Chromosomes by: Name’ (https://genomevolution.org/r/pmde).
5. To add synonymous substitution information to the dot plot, find the option to use CodeML under the Analysis Options tab and click on ‘Calculate syntenic CDS pairs and color dots: substitution rate(s)’. Start by selecting Synonymous (Ks) and click on Generate SynMap (https://genomevolution.org/r/pmdf). A SynMap showing the Ks value of each syntenic gene-pair and a histogram of the distribution of log10 transformed Ks substitutions will be generated. On SynMap2, the histogram can be generated for a given set of syntenic genes by selecting and dragging the cursor over any dotplot region. The same analysis can be performed for the Kn and Kn/Ks values. The analysis results can be downloaded by selecting the ‘click to view options’ next to ‘Download Results’, and then selecting the ‘Results with synonymous/non-synonymous rate values’.

#### SynMap results

**Figure 5.**
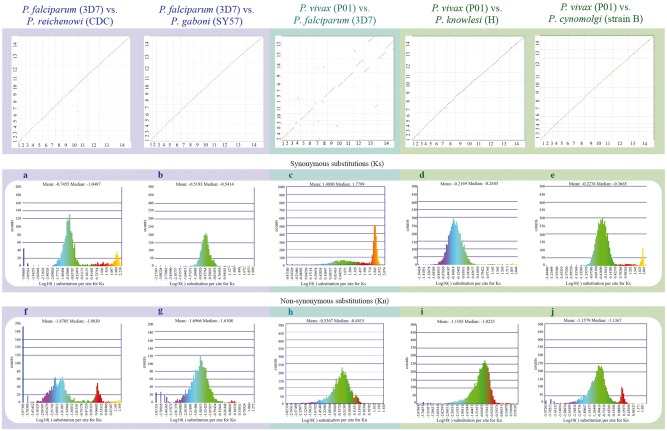
Syntenic dot plots between *P. vivax, P. falciparum* and related species*.* Top left: syntenic dot plot of pairwise comparisons in species with different levels of relatedness to *P. falciparum* (sister species, *P. reichenowi*; closely related species, *P. gaboni*). Top middle: syntenic dot plot of pairwise comparison between distantly related species *P. falciparum* and *P. vivax*. Top right: syntenic dot plot of pairwise comparisons in species with different levels of relatedness to *P. vivax* (sister species, *P. cynomolgi*; closely related species, *P. knowlesi*). Middle (**a–e**): histograms of Ks values for syntenic genes between species pairs: a *P. falciparum* vs. *P. reichenowi* (https://genomevolution.org/r/ljhj); b *P. falciparum* vs. *P. gaboni* (https://genomevolution.org/r/ljhl); c *P. falciparum* vs. *P. vivax* (https://genomevolution.org/r/ttfp); d *P. vivax* vs. *P. knowlesi* (https://genomevolution.org/r/lquk) and e *P. vivax* vs. *P. cynomolgi* (https://genomevolution.org/r/lquj). Bottom (**f–j**): histograms of Kn values for syntenic genes between species pairs: f *P. falciparum* vs*. P. reichenowi* (https://genomevolution.org/r/lsz2); g *P. falciparum* vs. *P. gaboni* (https://genomevolution.org/r/lsz5); h *P. falciparum* vs. *P. vivax* (https://genomevolution.org/r/ttft); i *P. vivax* vs. *P. knowlesi* (https://genomevolution.org/r/norf) and j *P. vivax* vs. *P. cynomolgi* (https://genomevolution.org/r/nore). Links to regenerate these analyses are in Supplementary File S2.

Broad-scale genome organization was largely maintained for the sister taxa (*Plasmodium cynomolgi*; https://genomevolution.org/r/lquj) and closely related species (*P. knowlesi*; https://genomevolution.org/r/lquk) to *P. vivax* (Figure 5). The same pattern was observed for the sister taxa (*Plasmodium reichenowi*; https://genomevolution.org/r/ljhj) and closely related species (*Plasmodium gaboni*; https://genomevolution.org/r/ljhl) to *P. falciparum* (Figure 5). On the other hand, several large-scale genome rearrangement events were evident when distantly related species (i.e. *P. vivax* and *P. falciparum*; https://genomevolution.org/r/ttfp) were compared (Figure 5). Although previous studies have hinted at a certain degree of organizational conservation between *Plasmodium* species (6, 22), these results clearly show that genome organization is largely dependent on the evolutionary relationships within the genus.

Species-specific substitution trends were characterized in closely to distantly related species (Figures 2 and 5) as a mean to assess relations between genome organization and evolution at the nucleotide level. Synonymous (Ks) and non-synonymous (Kn) substitution rates were calculated between syntenic gene pairs using CodeML. Although intra-clade variation was observed in both the simian clade and *Laverania* subgenus, in general, Ks and Kn values varied slightly more amongst parasites of the subgenus *Laverania* than in their simian clade counterparts (Figure 5a, b, d–g, i, j). The highest Ks (Figure 5c) and Kn (Figure 5h) values were found in comparisons between human-infective parasites [*P. vivax* and *P. falciparum;* ∼35 Mya. (18)].

The distribution of Ks values between *P. vivax* and *P. cynomolgi* [∼3.25–3.77 Mya. (18)] and between *P. vivax* and *P. knowlesi* [5.42–6.43 Mya. for Southern Asian parasites (18)], suggest that there are no considerable changes in mutation rates between these species at a genome-wide level. In contrast, differences in Ks and Kn values were more prevalent in comparisons between species of the *Laverania* subgenus, perhaps as a result of slightly older intra-clade divergence times [∼5.28–5.93 Mya. for *P. falciparum*/*P. reichenowi* (18) and ∼7–9 Mya. for *P. falciparum*/*P. gaboni* (19, 35)].

The high Ks and Kn values observed between *P. vivax* and *P. falciparum* likely reflect unique biological characteristics and species-specific adaptations. Genes coding for proteins with at least partly extracellular motifs are thought to evolve faster than other genes, even amongst closely related *Plasmodium* species (17). Such patterns are largely believed to be the result of the host immune system targeting extracellular peptides, forcing those genes to evolve faster to evade host defenses (17). Thus, the high Ks and Kn values seen between *P. vivax* and *P. falciparum* likely reflect both independent long-term responses to host–parasite interactions, and differences in CDS composition (17).

#### Syntenic path assembly

Although some model species have fully assembled genomes, for most groups in the tree of life only incomplete genome assemblies are available. CoGe’s syntenic path assembly (SPA) tool can help overcome some of the challenges posed by incomplete assemblies by ordering and orienting contigs from an incomplete assembly based on synteny to a reference genome (Supplementary File S3). Here, we reoriented and reorganized a complete (*P. falciparum*) and an incomplete (*Plasmodium inui*) genome assembly with SPA, using a *P. vivax* genome as a reference. In addition, the SPA can help make whole genome synteny of assembled genomes easier to visualize (Supplementary File S3). A diagram of the steps followed in this example workflow is included on Figure 4.

#### How to use SPA

1. Find the Display Options tab and select either SynMap2 or SynMap Legacy on the Visualizer Select menu from a previously generated SynMap (https://genomevolution.org/r/ttee). Locate the SPA tool and select it by clicking on the check mark next to ‘SPA?’ (SynMap2; https://genomevolution.org/r/weuq or SynMap Legacy https://genomevolution.org/r/tteh).
2. SynMap Legacy the SPA tool has additional visualization options. For instance, you can check the ‘Hide contigs without synteny?’ option to eliminate non-syntenic regions, which simplifies SynMap’s visualization (https://genomevolution.org/r/ttei).

#### SPA results

When comparing broad-scale genome organization between two complete assemblies (*P. vivax* vs. *P. falciparum*), reorientation with SPA aids in the interpretation of putative structural changes (e.g. identifying genome inversion). Alternatively, the organization of non-assembled contigs (*P. vivax* vs. *P. inui*) using SPA can be useful in identifying evolutionary complex regions (e.g. highly repetitive regions). It is, however, important to note that SPA can result in loss of identified structural changes as it enforces order by a reference genome.

#### GEvo

**Figure 6.**
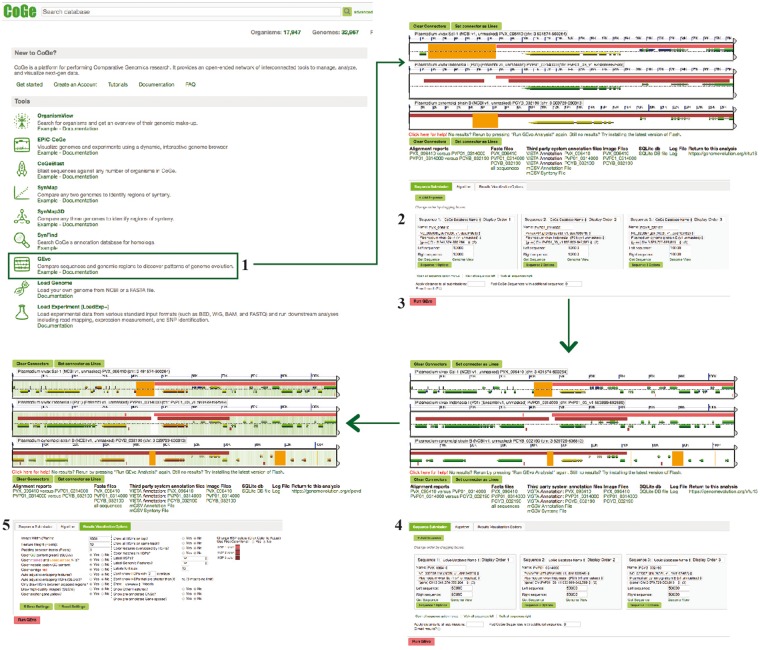
GEvo section of example Workflow 2. The displayed numbers match the steps indicated on the workflow section of the text. Links to regenerate these screen captures are provided within the step-by-step instructions found in the text.

Detailed microsynteny analyses of the regions identified by whole genome syntenic comparisons can aid in the identification of genome-specific characteristics or in finding discrepancies between assemblies. CoGe’s GEvo tool can be used to analyse and visualize local genomic organization and genomic features for microsynteny (differences in local genome organization are inferred by the collinear arrangement of homologous genes). This tool can be accessed via SynMap, by zooming in and selecting a gene-pair of interest or by searching specific Gene IDs in GEvo. Here, the *P. vivax* (Salvador-1 and P01) strains were compared with *P. cynomolgi* using SynMap and identified breakpoints were further evaluated using GEvo. A diagram of the steps followed in this example workflow is included on Figure 6.

#### How to use GEvo

1. Find GEvo on CoGe’s main page (https://genomevolution.org/coge/GEvo.pl).
2. Type a specific Gene ID on the ‘Name’ for each Sequence box (e.g. write PVX_095410 for Sequence 1 and PV01_0314000 for Sequence 2). Add another sequence box by clicking on the ‘+ Add Sequence’ button and type a third Gene ID (e.g. PCYB_032190 for Sequence 3).
3. Click on ‘Run GEvo’ with default parameters to display the local syntenic region between the compared genomes (https://genomevolution.org/r/pcvb).
4. Under the Sequence Submission tab, change the length of the microsyntenic region analysed by changing the ‘Left sequence’ and ‘Right sequence’ Genome View to 50000. For *Plasmodium* parasites this will amount to genomic regions containing ∼25 genes (https://genomevolution.org/r/pcvc).
5. Modify the graphical output display by selecting the ‘Only draw high-scoring sequence pairs (HSPs) between adjacent regions’, ‘Color GC content green’ and ‘Color wobble codon GC content’ options on the Results Visualization Options tab (https://genomevolution.org/r/pcvd).

#### GEvo results

**Figure 7.**
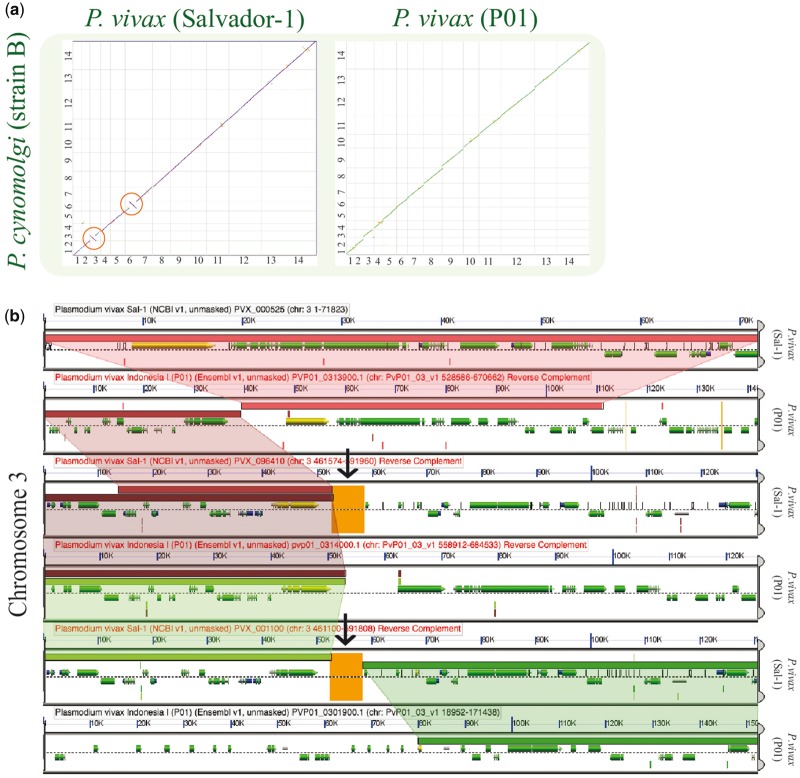
Analysis of breakpoint regions in *P. vivax* (Salvador-1). (**a**) SynMap pairwise comparisons of *P. vivax* strains Salvador-1 (https://genomevolution.org/r/lj12) and P01; (https://genomevolution.org/r/lquj) with *P. cynomolgi.* Orange circles show apparent inversions in *P. vivax* (Salvador-1). (**b**) Microsynteny analysis of the third chromosome’s shows breakpoint region close to region of poor sequence quality in orange (black arrow) (https://genomevolution.org/r/pho0). Wedges formed between adjacent genomes show regions of sequence similarity, a colinear set being used to identify syntenic blocks. Links to regenerate these analyses are in Supplementary File S2.

Synteny between the *P. vivax* (Salvador-1) genome and the *P. cynomolgi* was maintained with the exception of two previously reported (22) inversion events on Chromosomes 3 (∼20 000 bp) and 6 (∼50 000 bp). SynMap comparisons of *P. vivax* (P01) to *P. cynomolgi* revealed that *P. vivax* (P01) (https://genomevolution.org/r/lquj) lacked these inversion events (https://genomevolution.org/r/lj12) (Figure 7a). A microsynteny assessment of the breakpoint regions using GEvo showed syntenic regions of inverted genomic order on both Chromosome 3 (https://genomevolution.org/r/pho0) (Figure 7b) and in Chromosome 6 (https://genomevolution.org/r/phqb) in the *P. vivax* (Salvador-1) genome. Nonetheless, in both cases proximal regions of low sequence quality were observed only for *P. vivax* Salvador-1 (Figure 7b). Such regions are often filled with ‘N’ in the genomic assembly and are colored orange in GEvo. Given the improvements in sequencing and assembly technologies used in the P01 strain (36) with respect to those used on the Salvador-1 strain (17), it is likely that these regions represent assembly errors in the Salvador-1 genome.

### Workflow 3: finding multigene family members

Whole genome duplication and gene gain/loss events are prominent mechanisms for gene content variation (37). Evolutionary comparisons of gene content have been used to describe lineage-specific events [e.g. the degradation of metabolic pathways (38)], gene turnover rates between closely related species (39) and to study the role of duplications on evolutionary adaptation and innovation (40). CoGe’s tools can be used to explore these unique patterns in gene content evolution.

#### SynFind

SynFind (41) can identify the location of regions syntenic to a query gene, the syntenic depth (number of times a region is syntenic to target genome regions) and the number of genes in each syntenic region. Briefly, SynFind identifies homologous gene pairs using LAST (42) or LASTZ (43) for identifying sequence similarity. Later, a window of genes up and downstream from the query gene is selected by the researcher in which a minimum number of genes must be found to define a region as syntenic. The final syntenic score is based on the number of genes found within the window passing the minimum number of genes’ threshold. In addition, a research may a scoring function whereby matches within a window are collinear or just present (density).

**Figure 8.**
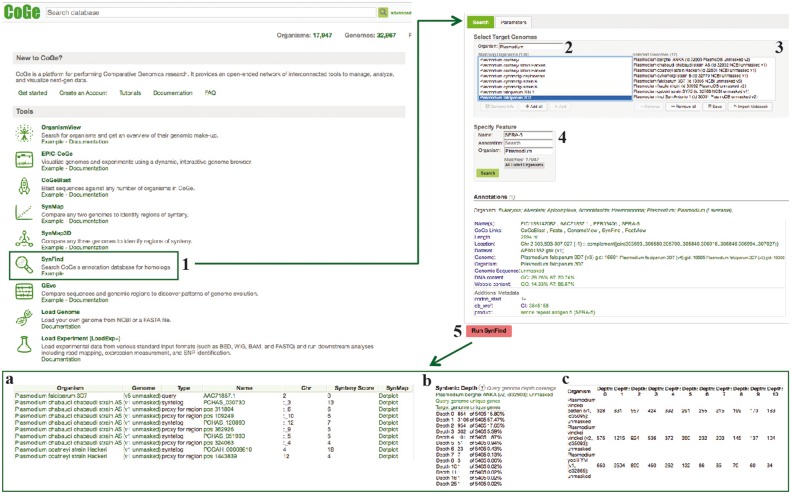
SynFind section of example Workflow 3. The displayed numbers match the steps indicated in the workflow section of the text. Screen capture of results from SynFind analysis: (**a**) summary table of syntelogs and genes proxy by regions; (**b**) syntenic depth table and (**c**) summary table of syntenic depth for all evaluated species. Links to regenerate these screen captures are provided within the step-by-step instructions found in the text.

These results can then be utilized to generate genome-wide lists of syntenic gene sets or be sent to GEvo for microsyntenic analysis. In *Plasmodium* spp., differences in gene content are often associated with changes in multigene family size and organization observable at the inter- and intra-specific levels (22, 44, 45). Here, we used two *Plasmodium*-specific multigene families [serine repeat antigen (SERA) (45) and cytoadherence-linked asexual gene (CLAG) (46)] as models for the analysis of multigene family evolution and gene content change. *Plasmodium falciparum* SERA-5 (PlasmoDB ID: PF3D7_0207600), a putative vaccine candidate (47) and *P. falciparum* CLAG-9 (PlasmoDB ID: PF3D7_0935800), a gene implicated in cytoadherence of infected erythrocytes (48) and solute transport (46) were used as family-specific gene queries. A diagram of the steps followed in this example workflow is included on Figure 8.

#### How to use SynFind

1. Find SynFind on CoGe’s main page (https://genomevolution.org/CoGe/SynFind.pl).
2. Type an organism’s name in the Select Target Genomes ‘Search’ box. Organisms and genomes with names matching the search term will be displayed on the Matching Organisms menu (e.g. type *Plasmodium*). Note that SynFind requires genomes to have CDS (protein CDS) structural annotations.
3. Select the genomes of interest and click on ‘+ Add’. The genomes will appear on the Selected Genomes menu [e.g. select *P. falciparum* 3D7 (id 19306 NCBI unmasked v5) from the list of available genomes]. Alternatively, all genomes of interest can be selected from any saved Notebook by clicking on ‘Import List’.
4. Type the Gene Name, Annotation or Organisms on the ‘Specify Features’ section and click on ‘Search’. All matches to the search term and the genome where they have been found will appear in a new menu. Select the relevant match and its reference Genome (e.g. type SERA-5 under ‘Name’ and *Plasmodium* under ‘Organism’). Change the SynFind general parameters (i.e. comparison algorithm) or synteny finding parameters (i.e. gene window size, minimum number of genes and maximum syntenic depth) before starting the analysis if needed.
5. Click on ‘Run SynFind’ to start the analysis (https://genomevolution.org/r/ohlf). Results can be selected and exported from the ‘Download’ menu.

#### SynFind results

SynFind identified a unique number of syntelogs (syntenic gene copies) and regional proxies (syntenic regions missing the query gene and thus potential evidence of duplication followed by loss) when *Pf*CLAG-9 (https://genomevolution.org/r/ohll) or *Pf*SERA-5 (https://genomevolution.org/r/ohlf) were queried (Supplementary File S4). At least one *Pf*CLAG-9 syntelog or regional proxy was found for all analysed *Plasmodium* species. In contrast, multiple *Pf*SERA-5 syntelogs or regional proxies were identified in each species, with some exceptions. These analyses show the distinctive evolutionary patterns of both families, with many SERA paralogs having conserved synteny while CLAG paralogs do not.

#### CoGeBLAST

**Figure 9.**
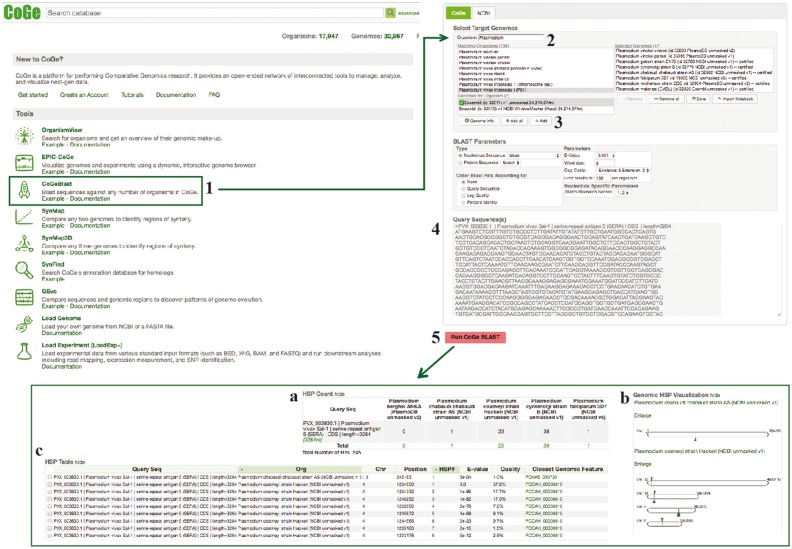
CoGeBLAST section of example Workflow 3. The displayed numbers match the steps indicated in the workflow section of the text. Screen capture of results from CoGeBLAST analysis: (**a**) HSP table; (**b**) genomic HSP visualization and (**c**) details of hits obtained for each evaluated species. Links to regenerate these screen captures are provided within the step-by-step instructions found in the text.

CoGeBLAST uses the BLAST suite of search algorithms (49) or LASTZ (43) to query any set of genomes in CoGe and further extends the base functionality of BLAST by incorporating useful genome visualizations into the search results. CoGeBLAST’s visualization can be used to identify patterns of gene organization (e.g. the organization of *Plasmodium* multigene families SERA and CLAG). In addition, CoGeBLAST results can be sent to GEvo for microsynteny analysis, enabling closer examination of local genome organization near query genes, as well as extracting the sequences of genes with significant BLAST hits for additional downstream analyses (e.g. inferring phylogenetic relationships). CoGeBLAST was used to perform sequence similarity searches across *Plasmodium* genomes and further explore differences in gene content. A diagram of the steps followed in this example workflow is included in Figure 9.

#### How to use CoGeBLAST

1. Find CoGeBLAST on CoGe’s main page (https://genomevolution.org/coge/CoGeBlast.pl).
2. Type a scientific name in the Organism ‘Search’ box. All genomes with names matching the search term will appear under the ‘Matching Organisms’ menu [e.g. type *Plasmodium* and select *P. vivax* Indonesia 1 (P01) Ensemble: (id 32811) v1 unmasked 24 214 674 nt]. Sets of genomes may be imported from any saved Notebook by clicking ‘Import List’.
3. Select all the genomes of interest and click ‘+ Add’. The genomes will appear in the ‘Selected Genomes’ menu.
4. Enter your query sequence in FASTA format (Supplementary File S5). Change the BLAST parameters before starting the analysis if needed (e.g. specifying nucleotide or protein query sequence).
5. Click on ‘Run CoGeBLAST’ (https://genomevolution.org/r/ohl6). Results can be selected and exported from the ‘Download’ menu.

#### CoGeBLAST results

**Figure 10.**
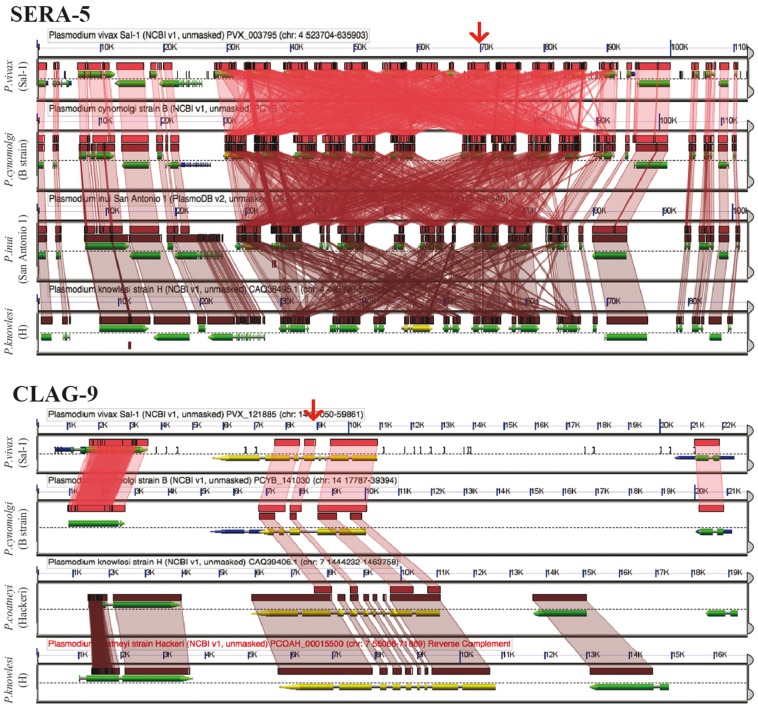
GEvo analysis using the CoGeBLAST’s output. Independent analyses are shown for the SERA (https://genomevolution.org/r/pee1) and the CLAG multigene families (https://genomevolution.org/r/z36c). Wedges formed between adjacent genomes show regions of sequence similarity in four *Plasmodium* species, a colinear set being used to identify syntenic blocks. Red arrow on top shows the location of the CLAG-9 and SERA-5 paralogs on *P. vivax* (Salvador-1). Note that SERA-5 exists in a tandem gene cluster, which results in having many overlapping regions of sequence similarity showing matches to each member of tandem gene cluster. Links to regenerate these analyses are in Supplementary File S2.

The number of significant CoGeBLAST (*E*-value < 1e^−40^, quality > 20%) hits varied across species for both *Pf*SERA-5 and *Pf*CLAG-9. These analyses can be reproduced using the sequences found in Supplementary File S5 and the parameters selected on the following CoGe links: *Pf*SERA-5 (https://genomevolution.org/r/ohl6) and *Pf*CLAG-9 (https://genomevolution.org/r/ohlj). Microsynteny analyses of the genome region containing four of the highest-ranking BLAST hits (HSP 1) for the SERA (https://genomevolution.org/r/pee1) and CLAG families (https://genomevolution.org/r/z36c) (Figure 10) demonstrated unique patterns of multigene family organization. Both families have had significant lineage-species contractions and expansions. However, SERA family members are arranged in tandem (44, 45); while not all CLAG members display a clustered genome distribution (46).

## Conclusions

The data presented herein is intended to serve as a demonstration of how CoGe’s tools and services can be used to assess genome-wide evolutionary patterns, further characterize sequenced genomes and perform different types of comparative genomic analyses. It should be noted that only a fraction of the tools and services available on CoGe have been covered here. Tools related to exploration of complex evolutionary patterns (e.g. codon change matrices) and features that allow group collaboration and data sharing have not been discussed. Furthermore, though we described CoGe tools using publicly available *Plasmodium* data; the instructions, tools and resources shown here are applicable in studies investigating any number of genomes from any species.

It is important to note that all analyses made in CoGe are reproducible, with links given to regenerate each analysis. Although CoGe is open for public and anonymous use for all publicly available genomes, for researchers that choose to get an account, CoGe will automatically track each analysis the researcher does and list them in their User page (Supplementary File S6). In addition, having a CoGe user account lets researchers add in their own data, keep them private and share them with collaborators. For computationally savvy researchers, CoGe also has a REST application programming interface that allows researchers to write programs to retrieve data, run analyses and integrate CoGe’s features into their programs. As more genomic data are generated, open computational platforms such as CoGe lets researchers easily manage and analyse their data without the needs to stand up the entire computational infrastructure required to support large-scale analyses.

## Declarations

### Ethics approval and consent to participate

Not applicable.

### Consent for publication

Not applicable.

### Additional resources

https://genomevolution.org/wiki/index.php/Main_Page and https://genomevolution.org/wiki/index.php/Using_CoGe_for_the_analysis_of_Plasmodium_spp.

## Supplementary data

Supplementary data are available at *Database* Online.

## Supplementary Material

Supplementary Data
