# Supplementary figures and images for: A tutorial of diverse genome analysis tools found in the CoGe web-platform using Plasmodium spp. as a model
Source: Database (Oxford). 2018 Apr 3;2018:bay030. doi: 10.1093/database/bay030 (PMC5887277; doi:10.1093/database/bay030)

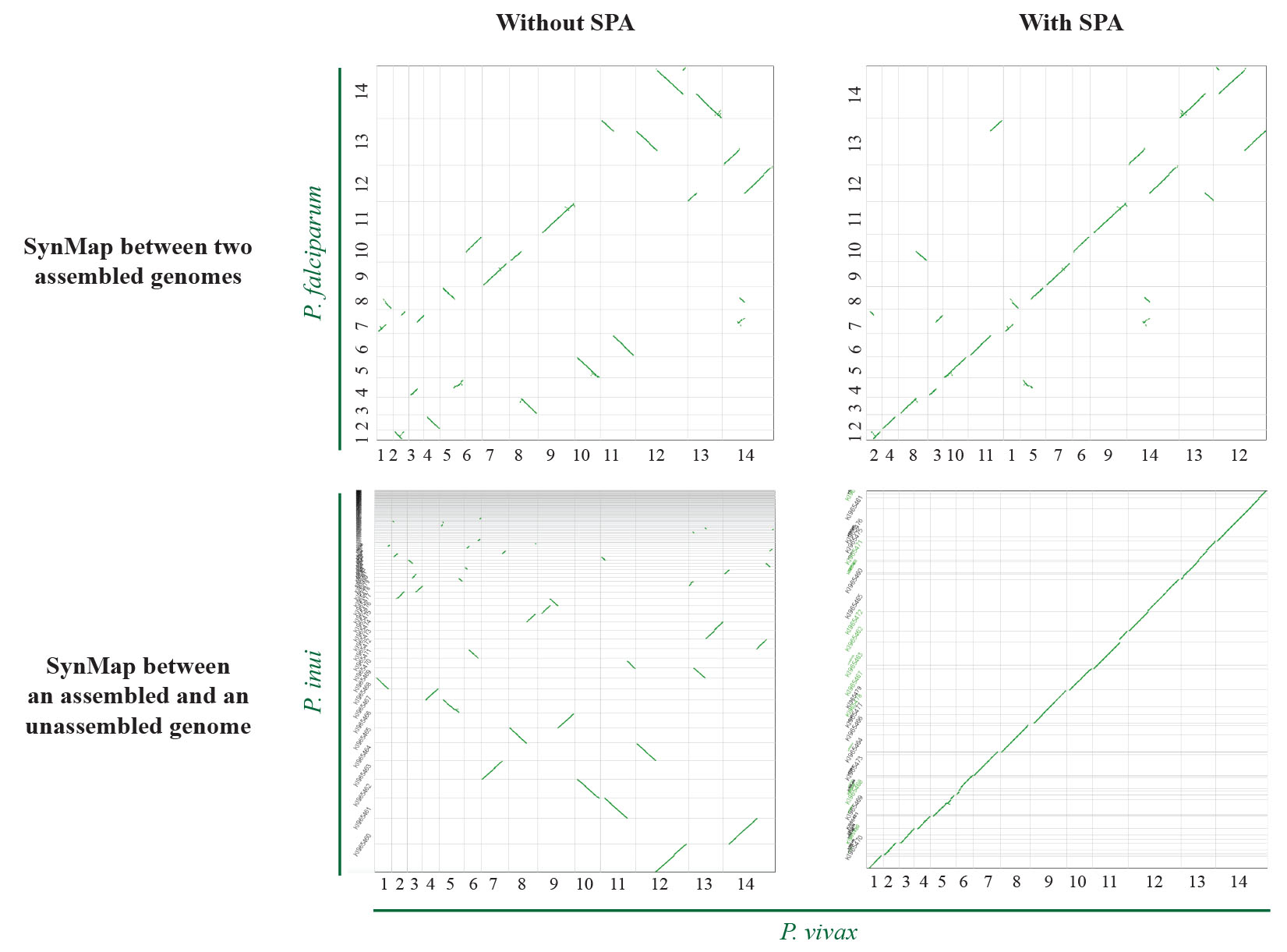

Supplement: Supplementary Data [file bay030_supp.zip › ACastillo_Supplementary_file_3_SPA.jpg]

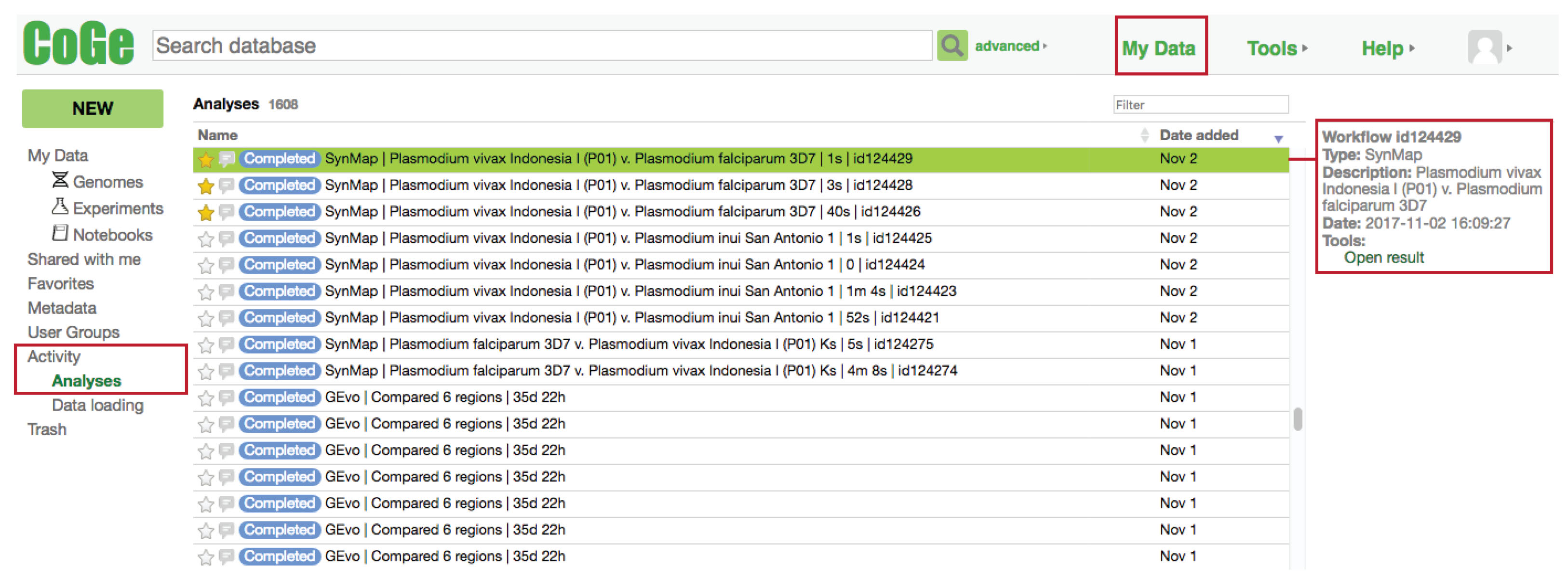

Supplement: Supplementary Data [file bay030_supp.zip › ACastillo_Supplementary_file_6_Users_analyses_list.jpg]
